# Need to Support Old-age Home Residents

**DOI:** 10.4103/0970-0218.40887

**Published:** 2008-04

**Authors:** Mukesh Kumar, RK Bansal, Manoj Bansal

**Affiliations:** Surat Municipal Institute of Medical Education and Research, Surat and UN Mehta Institute of Cardiology and Research Centre, Ahmedabad, Gujarat, India. E-mail: drrkbansal@gmail.com

Sir,

We had interviewed 68 elderly residing at four old-age homes in Surat city from October 2006 to January 2007 to explore their reasons for coming to stay at these homes. A total of 103 reasons were given by these 68 residents, which comprise neglected and living alone (36.9%); physical insecurity (16.5%); neglect after decreased income contribution (10.7%); lower medical care expenses and living costs in old-age homes as compared to living alone independently (9.7%); physical and verbal abuse and insults by daughter-in-law and son (8.7%); neglect by family due to high treatment costs and care (6.8%); poverty (5.8%); peaceful and spiritual life (1.9%); drug addict or abusive son (1%); schizophrenic son (1%) and belongings lost in the recent floods (1%).

The main reason that emerged was the unwillingness of the family to care for aged, which has been expressed through abuse, neglect and refusal to co-habit and care for them. This was pronounced in elderly who required extensive medical care, economically disadvantaged and those who had distributed their wealth to children. The elderly had reported of having felt unwanted and useless while living with their families. Forty-six percent had felt uncomfortable during their initial stay at old-age homes due to feeling of displacement from their neighbours, friends, relatives and family. They had stated that they had done their best for their children and society, and now they deserved reciprocation. Subsequently, 100% of them had felt better and comfortable, and 73.5% had opined that such homes were ideal settings for the aged as it is inevitable; abolishes everyday abuse; gives a sense of belongingness with fellow residents and provides better services. The qualities of services were better in paid homes, and this was reflected in their satisfaction levels. The discarding of this vulnerable group from the intergenerational family in the absence of adequate social networks and universal social security and left to increasing reliance on individual self, state and private institutions poses a grim future scenario for the elderly(1) who already face social problems and necessitating programmes to assist them to live fruitful lives as senior citizens.(2)

The study highlights the need to support the “Maintenance and Welfare of Parents and Senior Citizens Bill, 2007” in the Parliament, which mandates children and relatives to maintain senior citizens or pay monthly maintenance in cases of neglect and obliges the government to establish and support old-age homes; improve their healthcare provisions and introduce reverse mortgages and pension schemes.(3) The bill's enaction as law would greatly strengthen the constitutional and state commitments towards older people as mentioned in the National Policy on Older Persons(4) and would enable the elderly to reside with dignity in line with our Prime Minister's announcement on India's 60th Independence Day to launch programmes for pension, life, disability and health insurance cover to poor elderly.(5)
